# Influence of increased physical activity without body weight loss on hepatic inflammation in patients with nonalcoholic fatty liver disease

**DOI:** 10.1186/s12199-020-00857-6

**Published:** 2020-06-10

**Authors:** Fuminari Asada, Takuo Nomura, Atsushi Hosui, Masashi Kubota

**Affiliations:** 1 Research Center for the Health Promotion and Employment Support, Osaka Rosai Hospital, 1179-3 Nagasone, Kita-ku, Sakai City, Osaka 591-8025 Japan; 2 Department of Rehabilitation Sciences, Faculty of Allied Health Sciences, Kansai University of Welfare Sciences, 3-11-1 Asahigaoka, Kashiwara City, Osaka 582-0026 Japan; 3 Department of Gastroenterology and Hepatology, Osaka Rosai Hospital, 1179-3 Nagasone, Kita-ku, Sakai City, Osaka 591-8025 Japan

**Keywords:** Nonalcoholic fatty liver disease (NAFLD), Hepatic inflammation, Physical activity, Exercise guide, Metabolic equivalents (METs)

## Abstract

**Background:**

Physical activity (PA) that includes an accumulated exercise regimen that meets or exceeds a certain intensity reduces intrahepatic fat, leading to the improvement of nonalcoholic fatty liver disease (NAFLD) in afflicted patients. However, whether an increase in comprehensive PA, including activities of daily living, contributes to ameliorating the pathophysiology of NAFLD remains unclear. This study aimed to examine whether PA improves liver function in patients with NAFLD.

**Methods:**

The study included 45 patients with NAFLD who underwent follow-up examinations at least 6 months—but no later than 1 year—after their baseline examinations. The patients were interviewed about their daily activities and exercise habits to determine whether they had engaged in at least 3 metabolic equivalents (METs) per day during the previous 6 months; the quantity of PA, expressed in Ekusasaizu (Ex) units, was calculated as METs multiplied by hours. Patients who had achieved at least a 1-Ex increase in PA per week compared to baseline at the time of their follow-up interview (the PA increase group) were compared to those whose PA was the same or lower at the time of follow-up (the PA non-increase group).

**Results:**

There were no significant changes in all blood and biochemical parameters in the PA non-increase group at the time of follow-up when compared with baseline levels. In the PA increase group, aspartate aminotransferase, alanine aminotransferase, and γ-guanosine triphosphate levels were all significantly lower at follow-up than they were at baseline. Body weight did not change significantly from baseline to follow-up in both groups.

**Conclusions:**

In the present study, hepatic inflammation improvement was accompanied by increased PA but not decreased body weight. Increasing PA may be effective for the improvement of hepatic inflammation even without body weight loss. Our results indicate the effectiveness of PA monitoring for the management of NAFLD.

**Trial registration:**

UMIN-CTR, UMIN000038530

## Background

Nonalcoholic fatty liver disease (NAFLD) is a condition in which fatty liver occurs in the absence of alcohol-induced liver failure or other liver diseases and is normally diagnosed via histology and imaging [1]. NAFLD is observed in approximately 30% of all Japanese people and has a male predilection [2]. The survival rate of patients with NAFLD is lower than that of the general population [3], and the incidence of cardiovascular events is higher in the former than in the latter [4]. Most patients with NAFLD have an underlying condition such as obesity, diabetes, dyslipidemia, or hypertension. Weight gain, high aspartate aminotransferase (AST) and alanine aminotransferase (ALT) levels, low platelet counts, and high adipose tissue mass promote liver fibrosis [5]. Because physical activity (PA) improves obesity, blood glucose levels, and lipid concentrations while decreasing blood pressure [6], it is considered effective for managing NAFLD. A systematic review by Smart et al. revealed that exercise training improves cardiopulmonary function by decreasing intrahepatic fat and free fatty acid levels [7]. Purposely increasing PA to attain a certain minimum intensity may improve NAFLD by reducing intrahepatic fat. However, whether an increased comprehensive PA regimen comprising exercise and more vigorous daily activity affects the long-term pathophysiology of NAFLD remains unclear.

Obesity is the most important underlying etiology of NAFLD; in fact, visceral fat thickness is positively correlated with NAFLD severity [8]. As such, this study aimed to investigate the effect of PA on liver function in patients with NAFLD, with a focus on obesity.

## Methods

The study included 154 patients aged 20–85 years who attended the outpatient clinic of the Department of Gastroenterology, Osaka Rosai Hospital; could independently perform activities of daily living; and were given no exercise restrictions by their doctors. Patients were excluded from enrollment in this study if they tested positive for hepatitis virus infection, had a history of alcohol consumption (> 20 g/day), had difficulty performing exercise (owing to, for example, joint pain, kidney dysfunction, or heart disease), or had an underlying mental disorder. Each patient was provided an explanation of the study’s aim before providing written informed consent to participate and undergo the necessary baseline measurements. Among 108 patients diagnosed with NAFLD or nonalcoholic steatohepatitis (NASH) between March 2013 and December 2018 according to the diagnostic criteria issued by the Japanese Society of Gastroenterology [1], 45 who underwent follow-up examination at least 6 months after baseline—but no more than 1 year after—were examined. NAFLD was confirmed if diagnostic imaging revealed fatty degeneration of the liver in patients with no significant drinking history (i.e., an ethanol intake equivalent of less than 30 g/day and 20 g/day for men and women, respectively) and in whom viral liver disease was ruled out. A histological diagnosis of NASH was made when liver biopsy specimens fell into 1 of the following 2 clinicopathological categories [9]: (1) fatty change (regardless of severity) in hepatocytes combined with centrilobular ballooning and Mallory-Denk bodies or (2) fatty change in hepatocytes combined with centrilobular pericellular/perisinusoidal fibrosis or bridging fibrosis.

Data from hematological and biochemical examinations of blood samples drawn during outpatient clinic visits were used as the baseline. For body composition, limb muscle mass was determined using a composition analyzer (InBody720; Biospace Corp., Seoul, South Korea) [10] to calculate the skeletal mass index (limb muscle mass/height^2^). Waist circumference was directly measured at the level of the navel according to the method proposed by the Japanese Society for the Study of Obesity [11]. The same physical therapists were responsible for evaluating PA, physical function, and body composition, as well as for interviewing the patients about their diets and lifestyles. At that time, guidance to increase PA was provided based on the Exercise and Physical Activity Guide for Health Promotion 2006 (Exercise Guide 2006) issued by the Ministry of Health, Labour, and Welfare of Japan [12]. Each patient was asked to set their own goal for the quantum of physical activity, in consideration of their exercise habits. Then, the participant was informed of the extent of fat reduction that was possible by increasing physical activity for 6 months in accordance with an individualized plan (e.g., if a person with a weight of 70 kg walks briskly for 15 min every day for a period of 6 months, the increased caloric expenditure would theoretically lead to a fat reduction of approximately 1.8 kg).

This study was approved by the Research Ethics Committee of Osaka Rosai Hospital (approval number: 20130215) and is registered in the UMIN Clinical Trials Registry (UMIN000038530).

### Evaluation of PA and physical function

Each subject was interviewed to ascertain his or her successful engagement in moderate exercise and daily activities of 3 metabolic equivalents (METs) or higher during the previous 6 months according to the Exercise Guide 2006, and the Ekusasaizu (Ex) unit of quantity, which is calculated as METs × hours, was obtained [13]. Regular exercise was defined as that which was performed at least twice weekly for 30 min using the transtheoretical model [14].

Knee extension force (KEF), which is the isometric muscle force of the lower limbs at 90° of knee flexion, was determined using a handheld dynamometer with a fixing belt (μTas F-01; Anima Corp., Tokyo, Japan) [15]. The maximum KEF values of the right and left knees were averaged and normalized by body weight to obtain body weight rates (%) (KEF/body weight × 100). In the 30-s chair-stand test, subjects were observed for how many times they could repeat standing up from a 40-cm-high chair and sitting back down during a 30-s period while their arms were crossed over their chests [16].

### Evaluation of habitual behaviors

Data on eating behavior were obtained through individual interviews inquiring about diet during the preceding week using the recall method. Patients with a 20% higher or lower energy intake than that designated by the physician in charge (considered deviated calorie intake) were identified. The energy intake designated by the doctor was calculated with reference to light exertion (25–29 kcal/kg standard body weight), moderate exertion (30–34 kcal/kg standard body weight), and heavy exertion (≥ 35 kcal/kg standard body weight).

In terms of smoking habits, subjects who were daily or occasional smokers were considered present smokers, whereas those who never smoked or had not smoked during the previous 6 months were considered non-smokers.

### Statistical analysis

Patients whose weekly PA was at least 1 Ex greater at follow-up than it was at baseline were classified into the “PA increase” group and were compared with those who had an unchanged or decreased PA (the PA non-increase group). Qualitative variables of these groups at baseline were compared using the chi-square test, whereas quantitative variables were compared using the independent *t* test or the Mann–Whitney *U* test based on the Shapiro–Wilk test for normally distributed data. Qualitative variables at baseline and follow-up in each group were compared using the chi-square test, whereas quantitative variables were compared using the paired *t* test or the Wilcoxon signed-rank test based on the Shapiro–Wilk test for normally distributed data. Parameters that differed significantly between the groups were analyzed for their interactions by 2-factor repeated measures analysis of variance.

Stepwise multiple regression analysis was used to determine the amount of PA change corresponding to the amount of body weight change, and eating behavior (calorie intake as directed, 1; deviated calorie intake, 2), diabetes mellitus (absent, 0; present, 1), and smoking habits (absent, 0; present, 1) were included as covariates. Model 1 included the eating behavior at baseline while model 2 included that at follow-up. Diabetes mellitus and smoking habits were included in both analytic models. SPSS version 24 (IBM Corp., Armonk, NY, USA) was used for statistical analysis; the significance level was set at 5%.

## Results

The mean periods between baseline and follow-up were 295.3 ± 94.6 and 280.8 ± 96.1 days in the PA non-increase and PA increase groups, respectively, indicating no significant difference. The following data were shown as the mean value and standard error.

The total Ex value at follow-up in the PA non-increase group was 6.52 ± 1.0 Ex (3.57 ± 0.60 Ex for daily activities and 3.68 ± 0.95 Ex for exercise), showing a significant decrease from baseline (*P* < 0.001). In contrast, the total Ex value at follow-up in the PA increase group was 13.18 ± 1.5 Ex (4.30 ± 0.46 Ex for daily activities and 9.08 ± 1.71 Ex for exercise), showing a significant increase over the baseline value (*P* < 0.001). There were no significant intergroup differences with respect to patient characteristics (Table 1). Regarding eating behavior, none of the patients in either group had a caloric intake less than the designated level at baseline or follow-up; all patients with deviation had exceeded the designated caloric intake. Fourteen (70%) and 19 (76%) patients had deviated eating behaviors at follow-up in the PA non-increase and PA increase groups, respectively, indicating no significant difference from baseline in either group. Furthermore, there was no intergroup difference at follow-up; there was no significant change in smoking habits between baseline and follow-up.

**Table 1:** Baseline characteristics of patients with nonalcoholic fatty liver disease grouped by physical activity

Parameters	Units	Patients with no increased PA	Patients with increased PA	*P* value
NAFLD/NASH	*n*	7/13	14/11	0.231
Type 2 diabetes	Presence (%)	6 (30)	4 (17)	0.472
Sex	Male/female	10/10	11/14	0.769
Age	years	61.8 ± 2.5	56.5 ± 2.6	0.281
Body mass index	kg/m^2^	25.9 ± 1.1	26.6 ± 0.7	0.217
Body mass index ≥ 25	*n* (%)	9 (45)	15 (60)	0.377
Waist circumference	cm	89.0 ± 2.8	89.9 ± 2.3	0.392
Skeletal mass index	kg/m^2^	7.0 ± 0.2	7.1 ± 0.2	0.915
Physical activity and physical function				
PA for daily activities	Ex/week	4.6 ± 0.5	3.6 ± 0.3	0.189
PA for exercise	Ex/week	4.4 ± 1.1	3.3 ± 0.9	0.505
Total amount of PA	Ex/week	9.1 ± 1.2	7.0 ± 1.0	0.190
Regular exercise habits	Presence (%)	11 (55)	12 (48)	0.767
Knee extension force	kgf	32.7 ± 2.2	36.0 ± 3.2	0.429
%Knee extension force	%	49.2 ± 3.2	50.3 ± 2.8	0.810
CS30 test	*n*	21.8 ± 1.1	23.5 ± 1.1	0.307
Habitual behaviors				
Deviated eating behavior	*n* (%)	19 (76)	15 (75)	1.000
Current smoking habits	Presence (%)	2 (18)	5 (20)	0.437

Data are presented as *n* (%) or mean ± standard error

*NAFLD* nonalcoholic fatty liver disease, *NASH* nonalcoholic steatohepatitis, *PA* physical activity, *CS30* 30-s chair-stand test, *Ex* Ekusasaizu, metabolic equivalent × hour (unit of quantity)

**Fig. 1:**
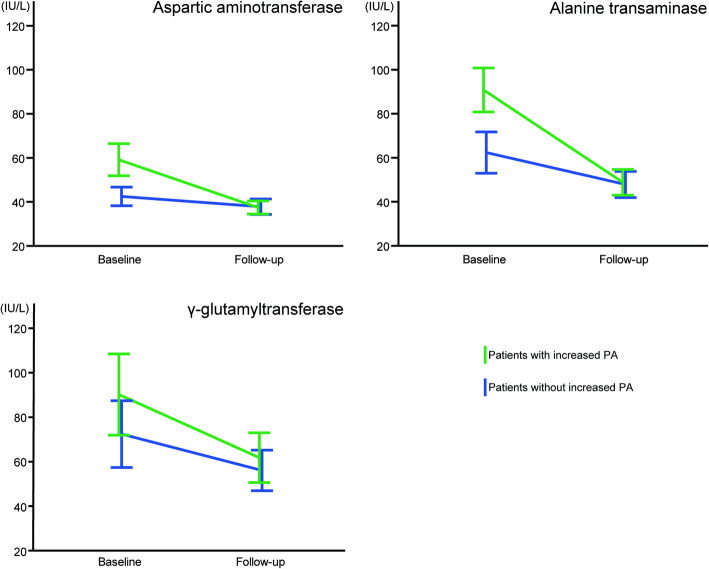
Levels of aspartic aminotransferase, alanine transaminase, and γ-glutamyltransferase between the physical activity increase and physical activity non-increase groups. Two-factor repeated measures analysis of variance. The green line indicates patients with increased physical activity (PA), whereas the blue line indicates patients without increased PA. The error bar presents the standard error in both groups. There was no significant effect of interaction in all laboratory findings

There were no significant changes in any blood or biochemical parameters measured at the time of follow-up when compared to baseline measurements in the PA non-increase group (Table 2). Conversely, there was a significant decrease in AST, ALT, and γ-guanosine triphosphate (γ-GTP) levels at follow-up in the PA increase group when compared to baseline. These results indicated a significant decrease in AST, ALT, and γ-GTP levels in both groups at follow-up as well as a tendency for AST, ALT, and γ-GTP to interact, although not significantly, in both groups (*F* = 2.287, *P* = 0.103; *F* = 3.909, *P* = 0.055; *F* = 0.117, *P* = 0.734, respectively) (Fig. 1). In stepwise multiple regression analysis, an increased PA was identified as a significant independent explanatory variable concerning the change in body weight in both analytic models (Table 3).

**Table 2:** Differences in clinical parameters in patients with or without increased physical activity

Parameters	Units	Baseline period	Follow-up period	*P* value
Patients without increased PA				
Body weight	kg	67.7 ± 3.8	68.3 ± 4.1	0.201
Aspartic aminotransferase	IU/L	41.9 ± 3.9	37.6 ± 3.4	0.238
Alanine transaminase	IU/L	59.2 ± 8.5	47.0 ± 5.8	0.067
Platelets	10^9^/L	210.6 ± 16.8	203.1 ± 16.8	0.629
FIB-4 index		2.10 ± 0.28	2.24 ± 0.36	0.459
FIB-4 index ≥ 2.67	*n* (%)	7 (35)	5 (25)	0.731
γ-Glutamyltransferase	IU/L	77.4 ± 15.8	58.3 ± 9.0	0.054
Lactate dehydrogenase	IU/L	209.6 ± 11.7	202.4 ± 8.2	0.279
Alkaline phosphatase	IU/L	269.1 ± 20.6	269.8 ± 24.7	0.944
Total bilirubin	mg/dL	0.66 ± 0.06	0.73 ± 0.06	0.360
Total cholesterol	mg/dL	190.0 ± 7.6	191.8 ± 8.3	0.716
Triglycerides	mg/dL	146.7 ± 18.1	179.3 ± 25.6	0.136
Albumin/globulin ratio		1.48 ± 0.04	1.46 ± 0.04	0.526
Patients with increased PA				
Body weight	kg	69.5 ± 3.3	67.8 ± 3.3	0.104
Aspartic aminotransferase	IU/L	61.4 ± 7.8	37.5 ± 3.0	0.004
Alanine transaminase	IU/L	95.3 ± 10.8*	49.5 ± 5.9	< 0.001
Platelets	10^9^/L	227.8 ± 12.8	231.6 ± 12.8	0.584
FIB-4 index		1.77 ± 0.24	1.56 ± 0.20	0.187
FIB-4 index ≥ 2.67	*n* (%)	5 (20)	4 (16)	1.000
γ-Glutamyltransferase	IU/L	89.1 ± 19.3	60.0 ± 11.5	0.015
Lactate dehydrogenase	IU/L	201.0 ± 7.5	194.4 ± 7.6	0.188
Alkaline phosphatase	IU/L	301.1 ± 33.6	284.9 ± 35.3	0.651
Total bilirubin	mg/dL	1.17 ± 0.32	0.93 ± 0.11	0.373
Total cholesterol	mg/dL	207.3 ± 9.1	205.9 ± 8.9	0.594
Triglycerides	mg/dL	186.7 ± 25.9	166.4 ± 31.8	0.292
Albumin/globulin ratio		1.35 ± 0.05	1.39 ± 0.06	0.268

Data are presented as *n* (%) or mean ± standard error

*FIB-4* fibrosis-4, *PA* physical activity

*P < 0.05, comparison between patients without increased PA and patients with increased PA during the baseline period

**Table 3:** Stepwise multiple regression analysis using body weight change as the objective variable

Explanatory variable	*β*	*t* value	*P* value	VIF
Model 1				
Change in total amount of PA	− 0.390	− 2.622	0.012	1.046
Baseline eating behavior	0.009	0.057	0.954	1.076
Presence of diabetes	0.111	0.750	0.458	1.037
Smoking habits	0.116	0.759	0.452	1.101
Model 2				
Change in total amount of PA	− 0.398	− 2.717	0.010	1.048
Follow-up eating behavior	0.157	1.078	0.288	1.030
Presence of diabetes	0.095	0.647	0.521	1.042
Smoking habits	0.098	0.669	0.507	1.044

*VIF* variance inflation factor, *PA* physical activity

## Discussion

In this study, patients with NAFLD were followed up for at least 6 months, but less than 1 year, and the specific parameters of individuals who engaged in increased PA were compared with those who did not undertake increased PA to investigate the effect of increased PA on liver function.

In a study of obese individuals, exercise therapy alone for over 12 weeks, but without dietary changes, reduced hepatic lipid levels despite no decrease in body weight [17]. Aerobic and resistive exercises improve blood glucose control, thereby improving insulin resistance and lipid metabolism. Therefore, such exercises are an established basic treatment for type 2 diabetes mellitus [18], although it may be difficult to maintain these exercise habits. In our previous study of 1442 patients with type 2 diabetes aged 30–87 years, only 26.9% exercised regularly [19]. It is also apparent that the level of daily activities is a major determinant of the differences in body weights between obese and non-obese individuals [20]; hence, exercise or daily activities may effectively manage NAFLD. There was a significant relation between the change in body weight and PA by the multivariate analysis in this study; however, the body weight did not significantly decrease by the univariate analysis in each group. Our study did not use a uniform exercise program, and PA intensity and duration varied among patients in the PA increase group. Increasing PA is important but not equivalent to performing a regular exercise for the improvement of hepatic inflammation without weight loss.

The “Specific Health Checkups” of Japan focus on metabolic syndrome and aim to prevent lifestyle-related diseases in 40–74-year-old individuals while guiding persons at high risk of developing such diseases [21]. The Specific Health Guidance is implemented with the aim of maintaining increased PA rather than confining exercise to a specific time frame during the day per the Exercise Guide 2006 [22]. In our study, an increase in non-standardized PA did not lead to body weight loss but improved hepatic inflammation, indicating the effectiveness of the Exercise Guide 2006 in the management of NAFLD. Interestingly, univariate analysis revealed a significant association between the decrease in laboratory findings reflecting hepatic inflammation, and the increase in PA; however, multivariate analyses revealed no significant interactions between each group. Attention should be paid for a better understanding of these results based on statistical analysis.

This study had several limitations. First, PA was evaluated in terms of the Ex value, and patients who had increased their PA by at least 1 Ex at follow-up relative to that at baseline were classified into the PA increase group. Although the mean increase was 6 Ex, the degree of effective increase in Ex remains unclear. In addition, patients were followed up for at least 6 months, and as a result, the end points in both groups were not unified. These differences may have resulted in endpoint variability. Second, the AST, ALT, and γ-guanosine triphosphate (γ-GTP) levels significantly decreased at follow-up in the PA increase group when compared to those at baseline. However, baseline ALT values were significantly higher in the PA increase group than in the PA non-increase group, and this may have influenced our results. Third, PA was evaluated according to the Exercise Guide 2016; therefore, it is unclear whether the actual exercises or the daily activities were responsible for greater body weight loss and improved hepatic inflammation. Furthermore, the evaluation and teaching methods based on the Exercise Guide 2006 were subjective. Therefore, it may be necessary to objectively quantify PA with a smart device. Fourth, the methodology of our study did not allow us to determine whether diet exerted an independent or synergistic effect on improving hepatic inflammation and body weight loss. Moreover, eating behaviors were evaluated based on the energy intake data from the previous week at both baseline and follow-up, as designated by the physician in charge. Therefore, the effects of eating behaviors during the period between baseline and follow-up are uncertain. Fifth, the study design prevented the evaluation of any synergistic effects of PA and diet. Further investigations are necessary to address these remaining issues.

## Conclusions

We noted significant improvements in ALT, AST, and γ-GTP levels in the PA increase group, which comprised subjects with increased PA by a mean of 6 Ex at follow-up compared to baseline. In contrast, while there were non-significant improvements in the blood and biochemical parameter measurements of the PA non-increase group, these differences were not as high as those observed in the PA increase group. Our data indicated that increased PA improves hepatic inflammation but does not decrease body weight in patients with NAFLD. Increasing PA may be effective for the improvement of hepatic inflammation without decreasing body weight. PA monitoring is important for the management of NAFLD.

## Data Availability

The datasets supporting the conclusions of this article are available from the corresponding author upon reasonable request.

## Abbreviations

ALT: Alanine aminotransferase
AST: Aspartate aminotransferase
Ex: Ekusasaizu (unit)
KEF: Knee extension force
MET: Metabolic equivalent
NAFLD: Nonalcoholic fatty liver disease
NASH: Nonalcoholic steatohepatitis
PA: Physical activity
γ-GTP: γ-Guanosine triphosphate
